# Supplementary material for: Exploring varicella zoster virus proteome for construction and validation of a multi-epitope based subunit vaccine using multifaceted immunoinformatics approaches
Source: PLoS One. 2025 Jun 24;20(6):e0324453. doi: 10.1371/journal.pone.0324453 (PMC12186983; doi:10.1371/journal.pone.0324453)
Supplement: S1 Table — (DOCX) [file pone.0324453.s001.docx]

Supplementary file (1)

Table (1): Showed the 73 proteins of VZV, their Entries and lengths of each proteins from uniprot database

| Protein  name | Entry (Accession Number) | length | Protein  name | Entry (Accession Number) | length |
| --- | --- | --- | --- | --- | --- |
| membrane protein UL56 | Q2PJ65 | 129 | envelope glycoprotein H | [Q6YPA5](https://www.uniprot.org/uniprotkb/Q6YPA5/entry) | 841 |
| membrane protein V1 | [Q6QCQ5](https://www.uniprot.org/uniprotkb/Q6QCQ5/entry) | 108 | tegument protein UL21 | [Q6QCT9](https://www.uniprot.org/uniprotkb/Q6QCT9/entry) | 541 |
| myristylated tegument protein CIRC | [A0A2R4QFN8](https://www.uniprot.org/uniprotkb/A0A2R4QFN8/entry) | 221 | envelope protein UL20 | [I2CNQ8](https://www.uniprot.org/uniprotkb/I2CNQ8/entry) | 222 |
| nuclear protein UL55 | [Q6QCQ3](https://www.uniprot.org/uniprotkb/Q6QCQ3/entry) | 179 | major capsid protein | [Q6QCL5](https://www.uniprot.org/uniprotkb/Q6QCL5/entry) | 1396 |
| multifunctional expression regulator | [Q6QCQ2](https://www.uniprot.org/uniprotkb/Q6QCQ2/entry) | 452 | capsid triplex subunit 2 | [Q6QCL4](https://www.uniprot.org/uniprotkb/Q6QCL4/entry) | 316 |
| envelope glycoprotein K | [Q6QCQ1](https://www.uniprot.org/uniprotkb/Q6QCQ1/entry) | 340 | DNA packaging terminase subunit 1 | [Q6QCL3](https://www.uniprot.org/uniprotkb/Q6QCL3/entry) | 747 |
| helicase-primase primase subunit | [Q6QCQ0](https://www.uniprot.org/uniprotkb/Q6QCQ0/entry) | 1083 | DNA packaging tegument protein UL17 | [Q6QCL2](https://www.uniprot.org/uniprotkb/Q6QCL2/entry) | 676 |
| tegument protein UL51 | [Q6QCP9](https://www.uniprot.org/uniprotkb/Q6QCP9/entry) | 259 | tegument protein UL16 | [Q6QCL1](https://www.uniprot.org/uniprotkb/Q6QCL1/entry) | 363 |
| deoxyuridine triphosphatase | [Q6QCP8](https://www.uniprot.org/uniprotkb/Q6QCP8/entry) | 396 | tegument protein UL14 | [Q4JQS9](https://www.uniprot.org/uniprotkb/Q4JQS9/entry) | 199 |
| envelope glycoprotein N | [Q0Q872](https://www.uniprot.org/uniprotkb/Q0Q872/entry) | 87 | tegument serine/threonine protein kinase | [Q6YP73](https://www.uniprot.org/uniprotkb/Q6YP73/entry) | 510 |
| tegument protein VP22 | [Q6QCP6](https://www.uniprot.org/uniprotkb/Q6QCP6/entry) | 302 | Deoxyribonuclease | [Q6QCT0](https://www.uniprot.org/uniprotkb/Q6QCT0/entry) | 551 |
| transactivating tegument protein VP16 | [Q6QCP5](https://www.uniprot.org/uniprotkb/Q6QCP5/entry) | 410 | myristylated tegument protein | [Q77NP3](https://www.uniprot.org/uniprotkb/Q77NP3/entry) | 81 |
| tegument protein VP13/14 | [G1K7V1](https://www.uniprot.org/uniprotkb/G1K7V1/entry) | 819 | envelope glycoprotein M | [Q77NP2](https://www.uniprot.org/uniprotkb/Q77NP2/entry) | 435 |
| **Tegument protein UL46 homolog** | [Q4JQW3](https://www.uniprot.org/uniprotkb/Q4JQW3/entry) | 661 | DNA replication origin-binding helicase | [G1K7Y8](https://www.uniprot.org/uniprotkb/G1K7Y8/entry) | 835 |
| thymidylate synthase | [Q4JQW2](https://www.uniprot.org/uniprotkb/Q4JQW2/entry) | 301 | helicase-primase subunit | [Q6QCK4](https://www.uniprot.org/uniprotkb/Q6QCK4/entry) | 771 |
| envelope glycoprotein C | [A8I3G1](https://www.uniprot.org/uniprotkb/A8I3G1/entry) | 560 | tegument protein UL7 | [Q77NP1](https://www.uniprot.org/uniprotkb/Q77NP1/entry) | 331 |
| envelope protein UL43 | Q4JQW0 | 406 | capsid portal protein | [Q6QCK2](https://www.uniprot.org/uniprotkb/Q6QCK2/entry) | 769 |
| DNA polymerase processivity subunit | [Q6QCN9](https://www.uniprot.org/uniprotkb/Q6QCN9/entry) | 408 | helicase-primase helicase subunit | [Q6QCK1](https://www.uniprot.org/uniprotkb/Q6QCK1/entry) | 881 |
| tegument host shutoff protein | [Q6QCW0](https://www.uniprot.org/uniprotkb/Q6QCW0/entry) | 455 | nuclear protein UL4 | [I2CNS4](https://www.uniprot.org/uniprotkb/I2CNS4/entry) | 196 |
| ribonucleotide reductase subunit 2 | [Q4JQV7](https://www.uniprot.org/uniprotkb/Q4JQV7/entry) | 306 | protein V57 | [Q6QCJ9](https://www.uniprot.org/uniprotkb/Q6QCJ9/entry) | 71 |
| ribonucleotide reductase subunit 1 | [Q4JQV6](https://www.uniprot.org/uniprotkb/Q4JQV6/entry) | 775 | nuclear protein UL3 | [Q6QCS0](https://www.uniprot.org/uniprotkb/Q6QCS0/entry) | 221 |
| capsid protein | [Q6QCN5](https://www.uniprot.org/uniprotkb/Q6QCN5/entry) | 483 | uracil-DNA glycosylase | [Q6QCJ7](https://www.uniprot.org/uniprotkb/Q6QCJ7/entry) | 305 |
| tegument protein UL37 | [Q6QCV6](https://www.uniprot.org/uniprotkb/Q6QCV6/entry) | 1038 | envelope glycoprotein L | [Q71S15](https://www.uniprot.org/uniprotkb/Q71S15/entry) | 159 |
| large tegument protein | [G9IV65](https://www.uniprot.org/uniprotkb/G9IV65/entry) | 2763 | ubiquitin E3 ligase ICP0 | [Q77NN8](https://www.uniprot.org/uniprotkb/Q77NN8/entry) | 467 |
| small capsid protein | [Q6QCN2](https://www.uniprot.org/uniprotkb/Q6QCN2/entry) | 235 | transcriptional regulator ICP4 | [P09310](https://www.uniprot.org/uniprotkb/P09310/entry) | 1310 |
| membrane phosphoprotein | Q6QCN1 | 269 | regulatory protein ICP22 | [P09255](https://www.uniprot.org/uniprotkb/P09255/entry) | 278 |
| DNA packaging protein UL33 | [Q6QCN0](https://www.uniprot.org/uniprotkb/Q6QCN0/entry) | 156 | virion protein US10 | [P09311](https://www.uniprot.org/uniprotkb/P09311/entry) | 180 |
| DNA packaging protein UL32 | [Q4JQU9](https://www.uniprot.org/uniprotkb/Q4JQU9/entry) | 585 | membrane protein US9 | [P09312](https://www.uniprot.org/uniprotkb/P09312/entry) | 102 |
| nuclear egress lamina protein | [I2CNP5](https://www.uniprot.org/uniprotkb/I2CNP5/entry) | 312 | serine/threonine protein kinase US3 | [P09251](https://www.uniprot.org/uniprotkb/P09251/entry) | 393 |
| DNA polymerase catalytic subunit | [Q6QCM7](https://www.uniprot.org/uniprotkb/Q6QCM7/entry) | 1194 | envelope glycoprotein I | [P09258](https://www.uniprot.org/uniprotkb/P09258/entry) | 354 |
| single-stranded DNA-binding protein | [Q6QCM6](https://www.uniprot.org/uniprotkb/Q6QCM6/entry) | 1204 | envelope glycoprotein E | [P09259](https://www.uniprot.org/uniprotkb/P09259/entry) | 623 |
| DNA packaging terminase subunit 2 | [Q6QCM5](https://www.uniprot.org/uniprotkb/Q6QCM5/entry) | 770 | virion protein US10 | [P09311](https://www.uniprot.org/uniprotkb/P09311/entry) | 180 |
| envelope glycoprotein B | [Q6YP71](https://www.uniprot.org/uniprotkb/Q6YP71/entry) | 931 | regulatory protein ICP22 | [P09255](https://www.uniprot.org/uniprotkb/P09255/entry) | 278 |
| phosphoprotein V32 | [Q4JQU3](https://www.uniprot.org/uniprotkb/Q4JQU3/entry) | 143 | transcriptional regulator ICP4 | [P09310](https://www.uniprot.org/uniprotkb/P09310/entry) | 1310 |
| capsid scaffolding protein | [Q6QCM2](https://www.uniprot.org/uniprotkb/Q6QCM2/entry) | 605 | nuclear protein UL24 | [Q6QCM0](https://www.uniprot.org/uniprotkb/Q6QCM0/entry) | 258 |
| **Major capsid scaffold protein** | [Q0Q846](https://www.uniprot.org/uniprotkb/Q0Q846/entry) | 302 | thymidine kinase | [G1K7Y3](https://www.uniprot.org/uniprotkb/G1K7Y3/entry) | 341 |
| DNA packaging tegument protein UL25 | [Q6QCU3](https://www.uniprot.org/uniprotkb/Q6QCU3/entry) | 579 |  |  |  |
