# Supplementary material for: Exploring varicella zoster virus proteome for construction and validation of a multi-epitope based subunit vaccine using multifaceted immunoinformatics approaches
Source: PLoS One. 2025 Jun 24;20(6):e0324453. doi: 10.1371/journal.pone.0324453 (PMC12186983; doi:10.1371/journal.pone.0324453)
Supplement: S2 Fig — (b): Capsid protein (c): Envelope glycoprotein C. (d): Envelope glycoprotein B (e): Capsid scaffolding protein. (DOCX) [file pone.0324453.s002.docx]

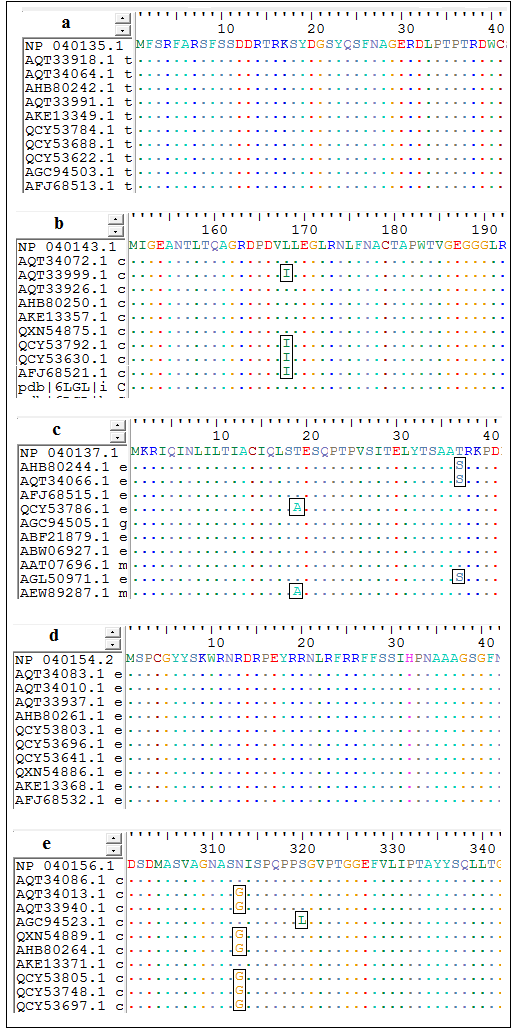


S2 Fig: showed the sequence alignment of the strain’s sequences for (a): **Tegument protein UL46 homolog**. (b): Capsid protein (c): Envelope glycoprotein C. (d): Envelope glycoprotein B (e): Capsid scaffolding protein. Letters within the squares showed mutated regions in the aligned sequence (unconserved regions), the dots indicated matches between the aligned sequences (conserved regions) using the BioEdit sequence alignment tool.
