# Supplementary material for: Exploring varicella zoster virus proteome for construction and validation of a multi-epitope based subunit vaccine using multifaceted immunoinformatics approaches
Source: PLoS One. 2025 Jun 24;20(6):e0324453. doi: 10.1371/journal.pone.0324453 (PMC12186983; doi:10.1371/journal.pone.0324453)
Supplement: Attachment — Submitted filename: Respond to reviewrs comments.docx [file pone.0324453.s003.docx]

Respond to reviewer’s comments

**Reviewer #1:**

 This study provided sufficient experimental evidence to support the conclusion. Overall, the results are solid and consistent. Several issues still need to be addressed before publication. The following questions may help the authors to improve the quality of their already outstanding manuscript, e.g. some important references are missing, such as PMID：38700327; 38075876; 37245745; 36094198 ; 35720422；PMID: 21835786 ；PMID: 38156274

Thank you very much for your comment

Based on your comment

We have cited the mentioned important references in our study

**Reviewer #2:**

The absence of any experimental validation significantly undermines the impact of the findings. Moreover, the manuscript relies heavily on computational tools without adequately addressing the limitations of these methods, particularly the potential discrepancies between predicted and actual immune responses in diverse human populations.  Although the in silico findings are promising, the manuscript lacks experimental validation of the predicted vaccine candidates. Including in vitro or in vivo data would strengthen the paper significantly.

Thank you very much for your comment

Based on your comment

We addressed the limitations of the in silico model, and we mentioned the potential discrepancies between predicted vaccine and actual immune responses in diverse human populations in the discussion section. This is an in silico study that based on prediction, thus the experimental validation of the predicted vaccine via in vitro or in vivo methods will be later studied.

The presentation of figures and tables is dense and difficult to interpret, and the paper lacks a detailed discussion on the real-world clinical implications and challenges of translating these findings into a viable vaccine. For instance, potential inaccuracies in molecular docking or population coverage analysis should be mentioned.

Thank you very much for your comment

Based on your comment

We improved the resolution of the figures and tables and made their interpretation easy to understand. Also we tried to connect our finding to the real world clinical implication of the vaccine and provided the inaccuracies and biases during the docking and population coverage analysis

Additionally, grammatical issues and overly complex sentence structures further hinder the readability of the manuscript.

Thank you very much for your comment

Based on your comment

We revised the language of the paper and corrected the grammatical errors and improve the readability of the paper

A more balanced discussion of the methodology’s limitations, along with experimental data, would greatly enhance the manuscript’s scientific rigor and overall clarity.

Thank you very much for your comment

Based on your comment

We made an improvement in the discussion section that we hope could balance with the methodology. Also the limitation of the of some tools were discussed

The phrase "The VZ is a human neurotropic disease" could be better stated as "VZV causes a neurotropic disease in humans."

Thank very much for your comment

Based on your comment

The phrase "The VZ is a human neurotropic disease" was changed to "VZV causes a neurotropic disease in humans."

• There are several instances where articles (e.g., "the" or "a") are missing. For example, "The vaccine was made of 615 amino acids" should read "The vaccine consists of 615 amino acids."

Thank very much for your comment

Based on your comment

We changed the (The vaccine was made of 615 amino acids" to be "The vaccine consists of 615 amino acids."

• The sentence "The vaccine's potential cloning in the pET28a (+) vector was assessed by in silico cloning that led to effective expression and translation" could be simplified for clarity. A suggestion: "In silico cloning was performed to assess the vaccine's expression in the pET28a (+) vector, showing promising results for translation."

Thank you very much for your comment

Based on your comment

The sentence "The vaccine's potential cloning in the pET28a (+) vector was assessed by in silico cloning that led to effective expression and translation" was changed to “In silico cloning was performed to assess the vaccine's expression in the pET28a (+) vector, showing promising results for translation."

• Some sections of the manuscript are repetitive, particularly in the methodology. Consolidating similar steps could improve the flow of the paper.

Thank you very much for your comment

Based on your comment

We have removed the repetitive parts in the manuscript and we tried to improve the flow of the paper to be readable

• The manuscript could improve its readability by ensuring all tables and figures are referenced properly within the text. A more structured and clear layout of data, especially in tables presenting epitopes, would enhance the presentation.

Thank you very much for your comment

Based on your comment

We improved the readability of the paper and tables and figures are referenced properly within the text.

While the manuscript utilizes a variety of bioinformatics tools, there is a heavy reliance on computational data without experimental validation. In silico predictions, while useful, are often prone to inaccuracies, and results may not translate effectively into in vivo or in vitro settings. The paper could improve by acknowledging the inherent limitations of computational models in vaccine development.

Thank you very much for your comment

Based on your comment

We added statements in the discussion section showing the limitation of this in silico study and we mentioned an in vitro and in vivo studies will be pursued to study the efficacy of the predicted vaccine in the future

• The concept of multi-epitope vaccine development is not entirely new, and similar approaches have been applied to other viruses. The novelty of this work lies in its specific focus on VZV, but the paper does not sufficiently highlight what sets this work apart from other similar immunoinformatics-based vaccine designs. More emphasis on novel contributions or innovative methodologies would strengthen the manuscript.
Thank you very much for your comment

Based on your comment

Your comment provides valuable feedback for us and highlights a critical area for improvement. The study focuses specifically on Varicella-Zoster Virus (VZV), which is a unique and relevant target. The paper focuses in the innovative computational methods used and the unique epitope selection strategies. Additionally, paper addresses existing gaps in VZV vaccine research and provide greater clarity and significance to the process of vaccination of VZV

• The manuscript lacks consistent formatting, particularly in the methodology and results sections. Some sections are overly detailed, while others lack sufficient explanation or clarity. For example, the subheadings under the methodology are sometimes too granular, making the text difficult to follow. A more streamlined structure with consistent formatting and balanced detail would improve readability.

Thank you very much for your comment

Based on your comment

We made consistent formatting of the manuscript, particularly in the methodology and results sections and balanced detail were made to improve readability.

• While population coverage is discussed, the manuscript does not thoroughly address how the predicted epitopes will perform across diverse populations with varying HLA types. The focus on global population coverage might overlook critical details about specific populations at higher risk for VZV or those with unique HLA alleles that might respond differently to the proposed vaccine. A deeper exploration of regional or ethnic differences in immune response would enhance the relevance of the findings.

Thank you very much for your comment

Based on your comment

We made discussion on the bases of epitope selections and their impact on the calculation of the population coverage. We mentioned in the discussion section that the exclusion of some rare alleles and the genetic diversity in specific ethnic groups might affect the population coverage prediction process. Also exclusion of epitopes that are weak but biologically relevant binders would result in incomplete population coverage predictions.

Several figures, such as those representing the 3D structures, docking results, and immune simulation outputs, lack sufficient explanation or visual clarity. Additionally, the tables summarizing the epitopes could be more user-friendly. For instance, some tables are densely packed with information, making them hard to interpret quickly. Visual aids such as graphs or color coding could help clarify key points.

Thank you very much for your comment

Based on your comment

We tried to improve the resolution of the figures. Also sufficient information about the figure was provided in the figure caption or in the result section concerning the figure

• The manuscript does not adequately compare its approach to alternative vaccine development strategies, such as using live-attenuated or recombinant vector vaccines. By discussing the advantages and disadvantages of different approaches in relation to the multi-epitope vaccine proposed, the authors could provide a more comprehensive view of how their strategy fits within the broader field of vaccine development.

Thank you for your valuable comment

Based on your comment

We compared the alternative vaccine development strategies, such as using live-attenuated vaccine and we mentioned their disadvantages in relation to the multi-epitope vaccine proposed in the introduction and the discussion sections

• Although many bioinformatics tools are used throughout the manuscript, some are not introduced in sufficient detail. The criteria for selecting particular tools (e.g., PSIPRED, I-TASSER) over others are not discussed, which weakens the rationale for the computational workflow. It would be beneficial to include a justification for each tool used and its specific relevance to the problem at hand.

Thank you very much for your comment

Based on your comment

We provided the sufficient justification details for the selected tools for the prediction process.

• The epitope selection process appears somewhat oversimplified. While antigenicity, allergenicity, and toxicity are considered, the authors do not address other important factors, such as the stability of the selected epitopes or potential cross-reactivity with human proteins in depth. These are critical concerns that could affect the safety and efficacy of the vaccine.

Thank you very much for your comment

Based on your comment

Epitopes selection criteria such as conservancy antigenicity, their role in population coverage, their allergenicity and toxicity, epitope length, the structural and solubility of the vaccine were all mentioned within the materials and methods

• Although the study is computational, the manuscript does not mention any ethical considerations related to future in vivo or in vitro experiments. For a study involving human immune responses and potential future clinical trials, a brief section discussing ethical concerns would be appropriate, particularly around the safety of testing multi-epitope vaccines in vulnerable populations.

Thank you very much for your comment

Based on your comment

We mentioned the precautions upon using the clinical trial in populations with pre-existing health conditions (e.g., malnutrition, immunosuppression) should be carefully studied. Also inadequate understanding of immune response variability may lead to adverse effects disproportionately affecting vulnerable groups in the conclusion section

• Sentences are often overly complex, leading to confusion. Simplifying sentence structures and avoiding long, compound sentences would improve clarity.

Thank you very much for your comment

Based on your comment

We shorten the long sentences and made them simple and easy to read
• Subject-verb agreement is inconsistent in some instances. For example, "The vaccine were made of 615 amino acids" should be "The vaccine was made of 615 amino acids."

Thank you very much for your comment

Based on your comment

We changed “The vaccine were made of 615 amino acids" to "The vaccine consists of 615 amino acids." Also this was revised all over the paper
• Several terms are introduced without definition or context, such as "GPGPG linkers." These should be explained briefly when first introduced for a broader audience.

Thank you very much for your comment

Based on your comment

We provided explanation of the terms upon their first appearance in the paper

**Reviewer #3:**

1. In the methods section, the authors should briefly explain the algorithms used in the used servers.

Thank you for your valuable comment

Based on your comment

We briefly explained the algorithms used in the used servers.

2. Please perform the molecular docking between T-cell epitopes and MHC molecules.

Thank you for your valuable comment

Based on your comment

We performed molecular docking between T-cell epitopes and MHC molecules. The docking molecules were provided in a new figure in the manuscript

3. In the manuscript, the authors should provide a logical reason for using this type of linkers in the structure of the vaccine by citing a valid reference.

Thank you very much for your comment

Based on your comment

We have provided the reasons for using this type of linkers and the adjuvant in the discussion section. Also we provided a valid reference
4. The authors should present the results of the "Molecular Dynamic Simulation" and "Immune Simulation" sections in more detail.

Thank you very much for your comment

Based on your comment

Some information was added in the figure caption or in the results and discussion sections about the molecular dynamic simulation
5. The authors should further discuss why the current vaccine constructs is more advantageous than the previous ones. How do you see the future steps for your vaccine? What are your next planned steps? Add a discussion regarding the next needed steps, Limitation, and etc.?

Thank you very much for your comment

Based on your comment

We added information why the current vaccine is more advantageous the previous vaccines. Also we mention the future of our designed vaccine and we mentioned the limitation of the future need steps. All these additions we stated in the discussion section

6. Please provide the MM-PBSA calculations of the simulated trajectory.
Thank you for your valuable comment

Based on your comment

We find this section is representative and sufficiently explain the free energy of binding according to the prodigy server. In addition the MM-PBSA is more commonly and accurately implemented in small molecule-macromolecular system interactions. However, in our case the epitope-protein interaction is very complex and contain several protein residues that cannot be implemented by MM PBSA and need computational resources.

7. Please explain why you selected the pET28a plasmid for cloning your vaccine construct.
Thank you for the valuable comment

Based on your comment

We provided the advantages of the pET28a (+) vector at the end of the in silico cloning section in materials and methods

8. Please revise the English language of your manuscript.
Thank you for the valuable comment

Based on your comment

We highly appreciate your comment

We made comprehensive English Language revision to the manuscript

9. For the richness of the article, the authors should compare their study with other vaccine design studies. For this, the authors please use the following studies and cite them.
<https://doi.org/10.2174/1573409919666230612125440>
<https://doi.org/10.1080/07391102.2023.2258403>
<https://doi.org/10.1007/s12033-023-00949-y>
<https://doi.org/10.1016/j.ijbiomac.2024.131517>

Thank you for your valuable comment

Based on your comment

The recommended references were cited in the manuscript

**Reviewer #4:**

1. English grammar and overall language should be improved.
Thank you for the valuable comment

Based on your comment

We highly appreciate your comment

We made comprehensive English Language revision to the manuscript

2. A complete spell check needs to be performed.

Thank you for the valuable comment

Based on your comment

We highly appreciate your comment and a comprehensive spelling errors revision to the manuscript is made

3. Reference numbering should follow a consistent pattern. For example, in lines 66 and 67, reference [2] is repeated. Instead of duplicating it, use it appropriately throughout the manuscript.
Thank you for the valuable comment

Based on your comment

We revised the reference numbering all over the manuscript

4. Line 71 lacks clarity and does not make sense.
Thank you for your comment

Based on your comment

We modified the sentence in line 71 to be meaningful

5. Lines 93–105 are unclear and do not contribute meaningfully to the scope of the manuscript.

Thank you for your valuable comment

Based on your comment

We have modified the section to be meaningful and related to the study. Also this section was strengthened by another reviewer and requires to add how the antiviral drugs are used to treat varicella

6. In line 116, the terms "in silico" and "reverse vaccinology" are mentioned together. These are two distinct fields and should be treated separately. "In silico" should also be italicized.
Thank you for your comment

Based on your comment

We have deleted the word (reverse vaccinology) and modify the sentence accordingly. We also italicized the *in silico* word

7. The introduction lacks relevant information about current prevention strategies for VZV, their limitations, and the advantages of using in silico methods.
Thank you very much for your comment

Based on your comment

The introduction and the discussion sections contain information about current prevention strategies for VZV, their limitations, and the advantages of using in silico methods

8. Neither the abstract nor the introduction includes details on which proteins were used in the research.
Thank you very much for your comment

Based on your comment

The data about the proteins that were used to predict the epitopes are mentioned in the material and methods in details. This is because according to the viral proteome analysis the targeted proteins were selected

9. The significance of using GPGPG and YAA linkers should be supported by relevant literature.

Thank you very much for your comment

Based on your comment

We have provided the reasons for using this type of linkers and the adjuvant in the discussion section. Also we provided a valid reference

10. In line 345, "table (1)" should be modified to "Table 1."
Thank you very much for your comment

Based on your comment

"table (1)" modified to "Table 1." Also modified for all tables in the study

11. In Section 3.3, “VZV strains retrieval, sequence alignments, and epitope conservancy analysis,” explain the discrepancies in the selection of strains for different proteins. For instance, “61 strains were retrieved for the tegument protein UL46 homolog, 46 strains for envelope glycoprotein B, 172 strains for envelope glycoprotein C, 28 strains for capsid protein, and 55 strains for capsid scaffolding protein.” Why were different numbers of strains retrieved for each protein?
Thank you very much for your comment

Based on your comment

A different number of strains were retrieved based on the sequence similarity and the length of the retrieved strains to the reference sequence of the elected proteins.

We added this sentence in the material and methods and the results sections

12. What was the reference strain used in this study? How were the strains selected?
Thank you very much for your comment

Based on your comment

The reference strain used in the study was the: Human alphaherpesvirus 3 (varicella zoster viral genome-Dumas strain) with 73 proteins that were retrieved in FASTA

13. The results section requires clearer language. For instance, in line 441, the sentence “For the secondary structure prediction, the vaccine construct approved 23.74%” needs to be restructured to convey the intended meaning more effectively.

Thank you for the valuable comment

Based on your comment

We highly appreciate your comment

We made a comprehensive grammar and spelling errors revision to the manuscript

14. In line 562, "TL4" should be replaced with "TLR4."
Thank you for the valuable comment

Based on your comment

We changed the TL4 to the correct for TLR4

15. The quality of all the figures is poor and needs to be improved.
Thank you for the valuable comment

Based on your comment

We tried to enhance the resolution of the figures to be clearer

16. In the discussion section, line 595, the phrase “The aimed of this study” should be corrected to “The aim of this study.”
Thank you for the valuable comment

Based on your comment

We changed the phrase “The aimed of this study” should be corrected to “The aim of this study.”

17. Current vaccination methods and their limitations should be discussed with references to relevant literature.
Thank you for your valuable comment

Based on your comment

We added data about the current vaccination methods and their limitations in the introduction and the discussion sections

18. The manuscript lacks critical scientific information on VZV and its treatment strategies.

Thank you for the valuable comment

Based on your comment

We added information about the virus and the antiviral drugs used for the treatment of	Varicella infection in the introduction section

**Reviewer #5:**

1.  A multitude of tools are employed to do the analysis. Incorporate the version and database version in the study and provide the citation as well.
Thank you for your valuable comment

Based on your comment

We incorporated the version and database version in the study and provided the citation as well

2. To enhance the methodology, review the research paper by Venkatesan, Amrit, et al. titled "An in silico approach for prediction of B cell and T cell epitope candidates against Chikungunya virus," published in Immunological Medicine 46.4 (2023): 163-174.

Thank you for your valuable comment

Based on your comment

We cited the above article in the methodology section
3. Parameters are not being used when utilizing the tools/webserver. I suggest adding parameters if different tools are used. Also, ensure that the parameters are being used as the default.
Thank you for your valuable comment

Based on your comment

We added information about the tools used in the study and we mentioned if a default parameters were used

4. The analysis of the multi-epitope vaccine assembly could be improved by providing more detailed information on the selection criteria for the epitopes with high population coverage scores.
Thank you for your valuable comment

Based on your comment

We added details information on the selection criteria for the epitopes with high population coverage scores

5. Additionally, it would be beneficial to elaborate on the specific roles and advantages of the chosen linkers (GPGPG and YAA) in terms of their impact on vaccine efficacy and stability.
Thank you for your valuable comment

Based on your comment

We provided more information about the linkers to and their role in the vaccine efficacy

6. discuss the implications of the six histidine tag on the purification process and any potential effects on the vaccine's immunogenicity would enhance the overall understanding of the assembly strategy.
Thank you for your valuable comment

Based on your comment

The information about the histidine tag was discussed in the discussion and results sections

7. To enhance the analysis, it would be beneficial to provide a more detailed explanation of the methodology used for calculating population coverage.
Thank you for your valuable comment

Based on your comment

We added detailed information about the population coverage and we presented the results in the form of a new figure reflecting the results of the population coverage

8. Additionally, including specific results or statistics from the population coverage analysis would strengthen the findings.

Thank you for your valuable comment

Based on your comment

We added information about the population coverage and we presented the results in the form of a new figure reflecting the results of the population coverage
9. The analysis of protein interactions during the docking process is crucial for understanding biological mechanisms. However, to enhance the clarity and depth of the analysis, it would be beneficial to include a more detailed explanation of the significance of the docking results, particularly how the interactions with TLR4 could influence vaccine efficacy.
Thank you for your comment

Based on your comment

We added more details and explanation of the significance of the docking results, particularly how the interactions with TLR4 could influence vaccine efficacy in the discussion section

10. Additionally, discussing the criteria for selecting the docking parameters and the rationale behind using specific servers like HADDOCK and PRODIGY would provide more context.
Thank you for your comment

Based on your comment

We provided the reason behind the criteria for selecting the docking parameters and the rationale behind using specific servers like HADDOCK and PRODIGY in the discussion section

11. Including comparative data or previous studies that highlight the effectiveness of the docking approach used could also strengthen the analysis.
Thank you for your valuable comment

Based on your comment

We added more data about the docking previous studies

12. Lastly, a discussion on potential limitations of the docking process and suggestions for future research directions would offer a more comprehensive view of the study's implications.
Thank you for your comment

Based on your comment

We added some limitation of the docking process of some software in the discussion section and why HADDOCK was preferred in this study

13. Discussing the implications of the population coverage results in relation to vaccine development or disease prevention could also add depth to the analysis
Thank you for your valuable comment

Based on your comment

We added information about the population coverage and we presented the results in the form of a new figure reflecting the results of the population coverage

14. In the conclusion, the text outlines the main key finding related to the proposed epitopes for a VZV vaccine and suggests future directions for additional investigation.

Thank you for your valuable comment

Based on your comment

We modified the conclusion and we have just outlined the main findings in our study and the future directions of our vaccine
